# Association Between Multidimensional Environmental Enrichment Domains and Intrinsic Capacity Among Older Adults

**DOI:** 10.1093/geroni/igaf122.2665

**Published:** 2025-12-31

**Authors:** Guanzhou Wang, Minqing Zhang

**Affiliations:** Duke University, Durham, North Carolina, United States; Duke University, Durham, North Carolina, United States

## Abstract

Environmental enrichment (EE), extensively studied in animal models, integrates cognitive, physical, and social stimulation to enhance functional health, but its translation to human aging research remains limited. Intrinsic capacity (IC), defined by the World Health Organization (WHO), provides a holistic framework for assessing functional health essential for independence in older age. This study examined associations and interactions between EE domains and IC among older adults. Using cross-sectional data from 5,454 older adults in the English Longitudinal Study of Ageing (ELSA), EE domains were assessed through self-reported physical activity (high/low), cognitive engagement (frequency of stimulating activities), and social engagement (frequency of social and community interactions). IC was classified into high, medium, or low based on WHO’s Integrated Care for Older People (ICOPE) guidelines. Ordinal logistic regressions, adjusted for age, sex, education, smoking, and alcohol use, were performed. Results indicated significant independent associations between each EE domain and higher IC levels: physical activity (OR = 1.35, 95% CI = 1.17–1.56, p<.001), cognitive engagement (OR = 1.21, 95% CI = 1.12–1.30, p<.001), and social engagement (OR = 1.07, 95% CI = 1.02–1.11, p=.002).A significant interaction between cognitive engagement and physical activity (OR = 0.77, 95% CI = 0.66–0.89, p<.001) indicated combined influences distinct from their individual effects. Interactions involving social engagement were not significant. Findings support translating EE concepts into human aging research, highlighting multidimensional lifestyle approaches, especially integrating physical and cognitive activities, to promote healthy aging. Future longitudinal studies and intervention trials are needed to confirm these findings and test the effectiveness of multidimensional EE interventions for optimizing intrinsic capacity and related health outcomes.

